# Exploiting Natural Language Processing to Unveil Topics and Trends of Traumatic Brain Injury Research

**DOI:** 10.1089/neur.2023.0102

**Published:** 2024-03-06

**Authors:** Mert Karabacak, Ankita Jain, Pemla Jagtiani, Zachary L. Hickman, Kristen Dams-O'Connor, Konstantinos Margetis

**Affiliations:** ^1^ Department of Neurosurgery, Mount Sinai Health System, New York, New York, USA.; ^2^ School of Medicine, New York Medical College, Valhalla, New York, USA.; ^3^ School of Medicine, SUNY Downstate Health Sciences University, New York, New York, USA.; ^4^ Department of Neurosurgery, NYC Health + Hospitals/Elmhurst, New York, New York, USA.; ^5^ Department of Rehabilitation and Human Performance, Icahn School of Medicine at Mount Sinai, New York, New York, USA.; ^6^ Department of Neurology, Icahn School of Medicine at Mount Sinai, New York, New York, USA.

**Keywords:** hot topic, natural language processing, research trends, TBI, topic modeling, traumatic brain injury

## Abstract

Traumatic brain injury (TBI) has evolved from a topic of relative obscurity to one of widespread scientific and lay interest. The scope and focus of TBI research have shifted, and research trends have changed in response to public and scientific interest. This study has two primary goals: first, to identify the predominant themes in TBI research; and second, to delineate “hot” and “cold” areas of interest by evaluating the current popularity or decline of these topics. Hot topics may be dwarfed in absolute numbers by other, larger TBI research areas but are rapidly gaining interest. Likewise, cold topics may present opportunities for researchers to revisit unanswered questions. We utilized BERTopic, an advanced natural language processing (NLP)-based technique, to analyze TBI research articles published since 1990. This approach facilitated the identification of key topics by extracting sets of distinctive keywords representative of each article's core themes. Using these topics' probabilities, we trained linear regression models to detect trends over time, recognizing topics that were gaining (hot) or losing (cold) relevance. Additionally, we conducted a specific analysis focusing on the trends observed in TBI research in the current decade (the 2020s). Our topic modeling analysis categorized 42,422 articles into 27 distinct topics. The 10 most frequently occurring topics were: “Rehabilitation,” “Molecular Mechanisms of TBI,” “Concussion,” “Repetitive Head Impacts,” “Surgical Interventions,” “Biomarkers,” “Intracranial Pressure,” “Posttraumatic Neurodegeneration,” “Chronic Traumatic Encephalopathy,” and “Blast Induced TBI,” while our trend analysis indicated that the hottest topics of the current decade were “Genomics,” “Sex Hormones,” and “Diffusion Tensor Imaging,” while the cooling topics were “Posttraumatic Sleep,” “Sensory Functions,” and “Hyperosmolar Therapies.” This study highlights the dynamic nature of TBI research and underscores the shifting emphasis within the field. The findings from our analysis can aid in the identification of emerging topics of interest and areas where there is little new research reported. By utilizing NLP to effectively synthesize and analyze an extensive collection of TBI-related scholarly literature, we demonstrate the potential of machine learning techniques in understanding and guiding future research prospects. This approach sets the stage for similar analyses in other medical disciplines, offering profound insights and opportunities for further exploration.

## Introduction

Traumatic brain injury (TBI) has emerged as a public health concern of significant global proportions because of its profound impact on mortality, disability, and economic burden.^1^ Recent data indicate that, worldwide, ∼69 million persons annually experience TBI, positioning it as the leading cause of death and disability arising from traumatic injuries.^2^ This escalating issue has sparked substantial research efforts directed toward understanding, preventing, and managing TBIs.

Over recent decades, there has been an impressive surge in TBI research output, mirroring the increase in TBI cases globally. The exponential growth in scholarly publications poses a significant challenge for researchers, given that they must dedicate considerable time to compile and interpret these findings. Traditional systematic and scoping reviews can require months to complete.^3,4^ For instance, Allen and Olkin underscored that even MetaWorks, an efficient firm specializing in meta-analyses, spent ∼1139 h completing 37 meta-analyses.^5^ Often, the time needed to complete these studies is far greater. These numbers highlight the urgency to devise more efficient methodologies for research synthesis.

The adoption of natural language processing (NLP) and topic modeling offers a promising avenue to streamline the process of consolidating academic literature.^6,7^ By applying these techniques, researchers can efficiently unveil latent themes within large and diverse literary data sets, providing valuable insights across numerous research domains. Moreover, topic modeling enables the identification of significant trends, burgeoning interests, and waning areas in medical research by monitoring the prevalence of specific topics over time.^8^ In essence, it substantially boosts the efficiency of scoping reviews, a known labor-intensive aspect of medical research. Given the vastness of academic publications and the richness of data they hold, the value of topic modeling in managing and interpreting this information is notably accentuated.

This study uses an NLP technique to investigate the publication landscape of TBI research, primarily focusing on identifying prevalent topics and trends within this field. The study aims to achieve two primary objectives: first, to elucidate the main themes in TBI research, and second, to assess the current popularity or decline of these topics. Further, a methodological objective is set to underscore the utility and potential of NLP in refining research syntheses, offering a more streamlined approach to dissect and comprehend the intricate landscape of academic literature in TBI research. This is key to helping authors choose appropriate journals for their work and helping journals assess the consistency of the work they are publishing.

## Methods

### Ethical approval

Because the current study focused on analyzing existing scholarly literature and did not involve human subjects or personal data, there was no requirement to seek approval from an institutional review board.

### Data source

The Scopus database was utilized to conduct a search using the keyword “traumatic brain injury” specifically within the “TITLE” and “KEY” fields. To ensure the relevance of the articles, search results were refined using filters, limiting the “Document type” to “Article” and “Review” categories, selecting “Journal” as the “Source type” and setting “English” as the language. Further, we included only those articles published after the year 1990 to prioritize contemporary research and developments in TBI research. The downloaded documents contained relevant metadata elements, such as document title, abstract, author name(s), year of publication, and citation count.

### Pre-processing

The data analysis process started with pre-processing to cleanse the downloaded data and prepare it for further analysis. Articles missing abstracts were excluded to maintain a comprehensive data set. A synthesized column was created by combining the title and abstract of each article, which allowed a complete content analysis with both of these elements. Citation counts were divided into quartiles (Q1, Q2, Q3, and Q4) to facilitate a better understanding of citation impact across various topics. Exploring the distribution of identified topics across different journals led to the identification of the 10 most prevalent journals, whereas others were grouped under the category of “Other” for simplicity in analysis. To gain a better understanding of individual author contributions, the first and senior authors were extracted from the overall author list. The 10 most frequently occurring first and senior authors were identified separately, and the rest were categorized as “Other” to maintain simplicity in interpretation.

### Topic modeling

To uncover latent patterns and identify the unique topics in our data set, we used an NLP approach called topic modeling. Specifically, we utilized *BERTopic*,^9^ a topic modeling technique that leverages *BERT* (Bidirectional Encoder Representations from Transformers) embeddings^10^ and *c-TF-IDF* (Class-based Term Frequency – Inverse Document Frequency) to generate dense clusters, resulting in easily interpretable topics while preserving important words in the topic descriptions. *BERTopic* builds upon *BERT*, a powerful pre-trained language model that has significantly revolutionized the field of NLP by enabling context-aware understanding and improved performance across various tasks. We utilized the *S-PubMedBert-MS-MARCO* model to obtain sentence embeddings.^11^ This is a Hugging Face *sentence-transformers* model fine-tuned specifically for information retrieval tasks in the medical text domain. The text was further processed by removing common, non-informative words, known as stop words, using the *NLTK* (Natural Language Toolkit) library after the embeddings were obtained.^12^ Custom stop words relevant to the TBI domain were also included to ensure that the extracted topics were informative and specific.

Based on exploratory work to determine ideal parameter settings, we set the “min_topic_size” to 200, because this threshold yielded topics that were substantive and logically grouped. This parameter determines the minimum number of documents that can be assigned to a topic—a higher value results in fewer topics overall. The topic probability refers to the likelihood that a document is assigned a particular topic based on its content. A high probability suggests a significant prominence of the topic within the document, whereas a low probability suggests minimal relevance or representation. By using these probabilities, the model handles cases where articles may relate to multiple topics by categorizing each document into the single most probable, dominant topic. Further, we utilized a probability threshold to identify outlier documents. Documents were designated as outliers if they had less than a 5% probability of fitting into any of the model's topics. This statistical cutoff allowed us to separate documents whose content did not closely match the primary topic categories discovered by the model.

Once the BERTopic model was fully trained, it produced a list of keywords as well as representative documents for each unlabeled topic. These representative documents served as prototypical examples that elucidated the underlying theme of each topic. The BERTopic framework automatically selects three of these representative documents per topic. Then, through careful manual examination of both the extracted keywords and representative documents, we assigned descriptive topic labels by consensus among all authors. This qualitative label curation process involved verifying keyword relevancy, scrutinizing representative document content, holding group discussions, and consolidating opinions to culminate in a single agreed-upon label per topic. Additionally, we created word clouds to provide an immediate visual depiction of the most salient terms associated with every topic. The word clouds offered a quick, intuitive snapshot of each topic's key terms. Through consensus among the authors, scrutiny of keywords and representative documents, and generation of word clouds, we were able to comprehensively describe and label the set of topics extracted by the trained BERTopic model.

We selected the top 10 most frequently occurring topics identified by the BERTopic model for more detailed analysis. These “top 10 topics” refer to the 10 most frequently occurring topics identified by the *BERTopic* model. The distribution of these top 10 topics across citation quartiles, journals, first authors, and senior authors was then examined. This analysis provided insights into the popularity and impact of the topics.

### Trend analysis

Following the methodology applied by Bittermann and Fischer,^13^ we used linear regression models to analyze trends within the identified topics. In this context, hot topics refer to areas exhibiting increasing attention in recent years, as indicated by positive linear slopes over the full time period analyzed or when focusing solely on the current decade. On the other hand, cold topics represent areas getting less emphasis over time, manifesting as negative slopes. Identification of hot and cold topics reveals shifting priorities rather than judging absolute volume of publications. We opted against incorporating non-linearity and multi-layer perceptrons into our analysis, primarily because of the risk of overfitting and the inherent complexity of our data set. As such, our focus remained on linear trends, which simplified our methodology and improved the interpretability of the results. This approach allowed us to capture key information about the temporal popularity of various topics while avoiding the challenges posed by overfitting and the complexity linked with non-linear models.

The trend modeling process involved extracting topic probabilities, publication years, and topic names from the data set. The topic probability refers to the likelihood that a document is assigned a particular topic based on its content. A high probability suggests a significant prominence of the topic within the document, whereas a low probability suggests minimal relevance or representation. Mean topic probabilities were calculated annually for each topic by aggregating the individual probabilities. Linear regression models were then trained for each unique topic, utilizing the mean topic probability as the dependent variable and the publication year as the independent variable. The slopes of the regression lines from these trained linear regression models allowed us to distinguish between hot and cold topics: Positive slopes indicated hot topics, whereas negative slopes pointed to cold topics.

The trend analysis was conducted in two phases. In the first phase, we examined overall trends from the inception up to the date the Scopus database was accessed, providing us with insights into the evolution of topics over time. In the second phase, we focused specifically on trends in the current decade (the 2020s), which enabled us to identify emerging topics and recent advancements in the field.

### Computational tools and libraries for data analysis

The computational analyses were performed using Python 3.1 in Google Colab. Libraries such as pandas and numpy were used for data manipulation and analysis, nltk for stop words removal, sentence-transformers and BERTopic for topic modeling, sklearn for regression analysis, and wordcloud for generating word clouds.

## Results

The field of TBI has grown immensely over the past 30 years. Interestingly, in PubMed, the first TBI article was published in 1891, then the second in 1941, followed by a handful to a hundred a year until the 1970s. In 1990, there were 279 published articles, and around the 2000s, this number really took off, reaching nearly 716 in the year 2000. This past year, in 2022, this number had jumped to 5351 articles.

Initially, a total of 62,181 documents were obtained from the search. After refining the search with criteria that included selecting only “Article” and “Review” as the document types, limiting the source type to “Journal,” setting the language to “English,” and filtering out the articles published before 1990, 12,690 documents were excluded. An additional 1670 documents were excluded because of missing abstracts. Consequently, 47,821 documents remained, of which 42,422 were successfully categorized into 27 distinct topics, highlighting the diverse nature of research within the field over the past 30 years. The remaining 5399 documents that were unable to be categorized represented 11.3% of the total; these were excluded as outliers because they did not align with any topical category.

**FIG. 1.**
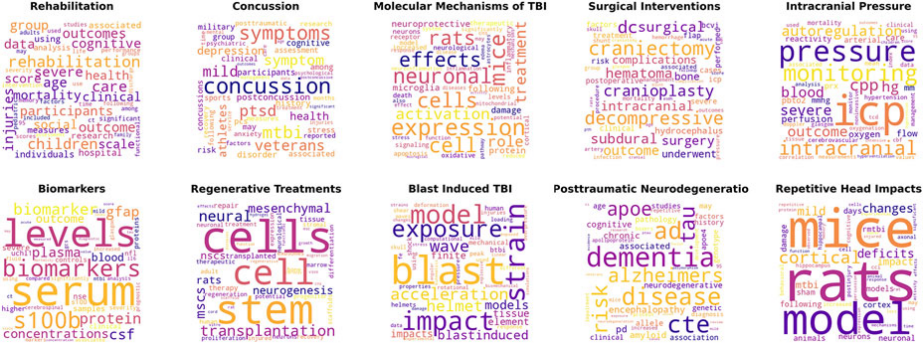
Word clouds of the top 10 topics.

The 10 most frequently occurring topics obtained from the BERTopic model were as follows: “Rehabilitation,” “Molecular Mechanisms of TBI,” “Concussion,” “Repetitive Head Impacts,” “Surgical Interventions,” “Biomarkers,” “Intracranial Pressure,” “Posttraumatic Neurodegeneration,” “Chronic Traumatic Encephalopathy,” and “Blast Induced TBI.” Table 1 presents these topics along with the rest, each characterized by a distinct set of keywords, while Supplementary Table S1 expands on this information by showcasing three representative documents that exemplify the thematic content of each topic. Additionally, for a more accessible and visual understanding of the topics, Figure 1 presents word clouds depicting the top 10 topics, while Supplementary Figure S1 extends this representation to the remaining topics. In these word clouds, the size of each keyword corresponds to its frequency, providing a concise summary of the main themes associated with each topic.

**Table 1.** Summary of the 27 Distinct Topics With Associated Keywords and Document Counts

Topic label	Keywords	No. of documents
Rehabilitation	rehabilitation, children, care, outcome, severe, outcomes, age, injuries, participants, data, group, cognitive, mortality, social, clinical, scale, health, score, hospital, individuals, research, using, measures, associated, analysis, significant, performance, factors, ct, functional, intervention, scores, pediatric, used, adults, following, included, severity, life, time, studies, use, among, 95, memory, may, assessment, groups, family, discharge	12,617
Molecular Mechanisms of TBI	expression, mice, neuronal, cells, cell, rats, effects, activation, treatment, role, neuroprotective, protein, following, microglia, damage, therapeutic, inflammatory, neurons, neurological, apoptosis, receptor, levels, oxidative, death, model, increased, cortical, signaling, significantly, also, mitochondrial, reduced, system, function, cns, may, potential, neuroinflammation, astrocytes, diseases, inflammation, mechanisms, effect, stress, response, pathway, edema, disease, activity, microglial	5240
Concussion	concussion, symptoms, mtbi, ptsd, mild, veterans, symptom, depression, athletes, health, postconcussion, disorder, history, participants, cognitive, concussions, military, stress, pcs, associated, among, risk, may, clinical, assessment, group, sports, posttraumatic, anxiety, reported, measures, recovery, research, following, psychiatric, time, factors, months, psychological, injuries, treatment, management, service, mental, care, postconcussive, significant, data, outcomes, age	4304
Repetitive Head Impacts	rats, mice, model, cortical, changes, mild, deficits, impact, mtbi, following, neurons, animals, cortex, days, neuronal, models, rmtbi, sham, damage, hippocampus, behavioral, cci, cell, increased, axonal, may, postinjury, cognitive, hippocampal, repetitive, loss, observed, expression, animal, rat, experimental, using, effects, motor, injured, mechanisms, function, time, cells, response, percussion, memory, protein, significant, activity	1801
Surgical Interventions	craniectomy, decompressive, dc, cranioplasty, hematoma, subdural, surgical, outcome, intracranial, surgery, complications, bone, underwent, hydrocephalus, postoperative, cases, bcvi, treatment, flap, risk, icp, performed, severe, clinical, factors, mortality, shunt, associated, hemorrhage, artery, outcomes, management, infection, group, procedure, pressure, skull, craniotomy, score, acute, rate, age, glasgow, complication, cranial, sdh, blunt, asdh, pth, followup	1696
Biomarkers	serum, levels, biomarkers, s100b, biomarker, protein, csf, gfap, plasma, concentrations, outcome, blood, samples, severe, nse, uchl1, clinical, higher, controls, proteins, nfl, s100, severity, significantly, glasgow, mtbi, ct, compared, increased, cerebrospinal, markers, fluid, analysis, associated, mild, using, gcs, il6, prognostic, diagnostic, tau, acute, concentration, may, level, scale, value, score, mortality, measured	1487
Intracranial Pressure	icp, pressure, monitoring, intracranial, cpp, autoregulation, outcome, severe, blood, hg, perfusion, mm, oxygen, flow, care, pbto2, reactivity, mmhg, arterial, prx, mortality, hypertension, mean, data, cerebrovascular, management, associated, clinical, oxygenation, tissue, noninvasive, values, cbf, using, analysis, tcd, index, monitor, measurements, glasgow, doppler, used, correlation, hyperventilation, score, outcomes, intensive, children, hypotension, 95	1430
Posttraumatic Neurodegeneration	dementia, disease, cte, ad, tau, risk, alzheimers, apoe, encephalopathy, pd, amyloid, chronic, cognitive, associated, neurodegenerative, allele, pathology, association, genetic, clinical, factors, studies, may, genotype, history, age, apoe4, diagnosis, apolipoprotein, protein, repetitive, boxers, increased, mice, 95, impairment, development, neurodegeneration, polymorphism, research, review, ptau, onset, mild, ci, veterans, cases, exposure, factor, evidence	1216
Blast Induced TBI	blast, strain, impact, model, exposure, acceleration, wave, helmet, blastinduced, models, impacts, tissue, finite, element, mechanical, btbi, human, shock, using, helmets, peak, properties, rotational, fe, loading, response, shear, skull, used, deformation, injuries, pressure, computational, different, waves, overpressure, data, experimental, damage, angular, biomechanical, linear, simulations, stress, exposed, changes, explosive, mild, strains, kinematics	1091
Regenerative Treatments	cells, stem, cell, transplantation, neural, mscs, mesenchymal, neurogenesis, rats, nscs, transplanted, tissue, therapy, repair, regeneration, neurons, therapeutic, human, treatment, adult, differentiation, proliferation, neurological, bone, potential, exosomes, recovery, stromal, progenitor, neuronal, injured, marrow, expression, scaffolds, regenerative, vitro, functional, diseases, scaffold, vivo, cns, cord, hydrogel, effects, function, msc, migration, growth, rat, nervous	1077
Diffusion Tensor Imaging	diffusion, matter, imaging, white, dti, fa, tensor, mri, mtbi, anisotropy, mild, fractional, volume, axonal, corpus, callosum, changes, dai, diffusivity, wm, regions, controls, lesions, group, tracts, resonance, magnetic, using, tract, tractography, diffuse, integrity, left, atrophy, mean, compared, showed, abnormalities, cognitive, subjects, analysis, outcome, months, control, right, associated, significant, values, findings, microstructural	1059
Coagulation	transfusion, coagulopathy, platelet, vte, hemorrhage, mortality, risk, blood, bleeding, txa, coagulation, intracranial, associated, tranexamic, prophylaxis, ich, venous, antiplatelet, anticoagulation, 95, acid, use, outcomes, ci, thromboembolism, admission, vs, outcome, progression, early, clinical, group, score, severe, dvt, ratio, warfarin, analysis, red, rfviia, time, ct, therapy, included, phi, received, compared, inr, higher, level	938
Cerebral Metabolism	glucose, lactate, blood, metabolism, microdialysis, pressure, resuscitation, rats, levels, metabolic, animals, increased, shock, significantly, group, hyperglycemia, perfusion, fluid, mm, arterial, outcome, groups, severe, effects, following, tissue, glutamate, hemorrhagic, energy, compared, pyruvate, oxygen, hg, treatment, control, cortical, increase, ratio, concentrations, flow, model, hs, may, map, associated, mean, vs, higher, edema, clinical	903
Genomics	expression, bbb, cns, diseases, cells, role, gene, genes, cell, barrier, mirnas, evs, hmgb1, therapeutic, calpain, complement, activation, neuronal, system, bloodbrain, inflammatory, exosomes, potential, extracellular, mice, disease, proteins, neurological, protein, nervous, target, immune, rna, vesicles, pathway, disorders, central, signaling, noncoding, molecular, rnas, inflammation, may, glioma, endothelial, also, micrornas, mechanisms, neuroinflammation, development	866
Posttraumatic Seizures	epilepsy, seizures, seizure, pte, eeg, posttraumatic, risk, levetiracetam, antiepileptic, phenytoin, pts, epileptogenesis, prophylaxis, epilepticus, early, ceeg, clinical, treatment, drugs, epileptic, incidence, monitoring, aeds, associated, factors, status, 95, drug, lev, development, studies, ci, pnes, age, may, data, children, continuous, cohort, se, aed, models, use, review, common, nonconvulsive, severe, diagnosis, care, electroencephalography	800
Neuroimaging	imaging, mri, magnetic, resonance, ct, neuroimaging, spect, clinical, mr, lesions, spectroscopy, naa, tomography, findings, images, techniques, swi, perfusion, using, proton, image, mrs, mild, outcome, computed, data, pet, abnormalities, segmentation, used, studies, detection, review, lesion, diagnosis, emission, may, acute, changes, normal, metabolic, applications, information, analysis, matter, evaluation, compared, scans, use, also	721
Biopsychosocial Factors	alcohol, use, substance, ipv, history, among, health, violence, women, abuse, risk, prevalence, lifetime, mental, associated, individuals, homeless, population, screening, drinking, injuries, offenders, bac, research, reported, suicide, participants, sample, 95, homelessness, intoxication, psychiatric, higher, criminal, ci, intimate, partner, age, severity, group, bal, studies, outcomes, data, tbis, association, compared, childhood, factors, violent	638
Brain Connectivity	connectivity, network, functional, mtbi, networks, regions, dmn, controls, mild, fmri, structural, cortical, restingstate, imaging, healthy, cognitive, frontal, cortex, right, magnetic, resting, ptsd, left, changes, group, default, meg, using, control, motor, resonance, associated, neural, gyrus, within, symptoms, thickness, analysis, mode, graph, mri, task, subjects, may, fc, state, cingulate, prefrontal, compared, alterations	591
Sensory Functions	visual, vestibular, mtbi, dizziness, vision, balance, eye, mild, symptoms, oculomotor, auditory, ocular, rehabilitation, vertigo, dysfunction, hearing, bppv, group, postural, participants, may, test, deficits, subjects, pupillary, measures, pupil, assessment, control, clinical, individuals, testing, controls, movements, tests, function, photophobia, performance, sensory, positional, blast, following, concussion, light, significant, reading, tracking, using, veterans, associated	589
Posttraumatic Neuroendocrine Dysfunction	pituitary, hypopituitarism, hormone, gh, deficiency, ghd, hyponatremia, cortisol, dysfunction, growth, endocrine, severe, hormonal, serum, levels, sodium, replacement, insipidus, hypernatremia, insufficiency, adrenal, anterior, may, acute, diabetes, deficiencies, function, neuroendocrine, common, hypogonadism, testosterone, diagnosis, months, prevalence, clinical, axis, associated, test, following, normal, acth, siadh, studies, syndrome, secretion, mortality, hormones, therapy, one, treatment	583
Mental Status	consciousness, state, eeg, mcs, doc, conscious, recovery, vegetative, minimally, coma, clinical, disorders, crsr, functional, agitation, rehabilitation, outcome, severe, qeeg, wakefulness, unresponsive, vs, potentials, uws, evoked, studies, scale, may, cognitive, using, showed, treatment, assessment, data, analysis, scalerevised, somatosensory, syndrome, prognosis, evidence, seps, months, power, diagnosis, motor, used, one, review, cortical, connectivity	520
Temperature Effects	hypothermia, temperature, cooling, therapeutic, fever, normothermia, hyperthermia, outcome, group, severe, th, trials, hypothermic, body, rewarming, clinical, treatment, effects, effect, rats, ttm, normothermic, management, mortality, studies, moderate, induced, hours, mild, intracranial, controlled, outcomes, may, data, control, therapy, randomized, care, arrest, cardiac, pressure, neurological, core, trial, icp, temperatures, significantly, groups, review, use	500
Posttraumatic Sleep	sleep, insomnia, disturbances, disturbance, sleepwake, quality, sleepiness, disorders, fatigue, daytime, osa, ptsd, veterans, actigraphy, mtbi, apnea, symptoms, participants, associated, following, treatment, disorder, posttraumatic, pain, mild, may, depression, swd, cognitive, problems, narcolepsy, hypersomnia, psqi, polysomnography, outcomes, severity, poor, light, rehabilitation, wake, time, subjective, measures, individuals, clinical, research, common, severe, group, anxiety	441
Brain Stimulation	stimulation, rtms, tdcs, transcranial, dbs, treatment, magnetic, group, vns, consciousness, tms, effects, neuromodulation, recovery, cognitive, electrical, motor, nerve, repetitive, studies, neurofeedback, noninvasive, direct, deep, current, disorders, clinical, review, vagus, coma, sensory, symptoms, cortex, sessions, sham, improvement, auditory, control, pain, changes, tremor, potential, memory, doc, significant, prefrontal, depression, trial, therapy, randomized	386
Hyperosmolar Therapies	mannitol, hypertonic, saline, hts, icp, fluid, resuscitation, intracranial, pressure, sodium, infusion, therapy, hyperosmolar, severe, use, mortality, hs, administration, trials, solutions, group, care, hypertension, serum, treatment, bolus, albumin, edema, trial, fluids, blood, received, osmolality, crystalloids, management, compared, effects, shock, volume, acute, outcome, crystalloid, solution, used, clinical, hg, randomized, 20, review, osmotic	314
Posttraumatic Headache	headache, pth, pain, posttraumatic, headaches, migraine, persistent, chronic, mild, ppth, treatment, common, mtbi, symptoms, associated, reported, veterans, concussion, symptom, may, studies, following, compared, allodynia, review, acute, clinical, controls, participants, severity, attributed, factors, soldiers, history, frequency, primary, characteristics, healthy, migrainelike, medication, months, prevalence, disorders, central, risk, group, one, individuals, phenotype, among	313
Sex Hormones	progesterone, estrogen, rats, e2, neuroprotective, effects, prog, treatment, steroids, hormones, levels, female, expression, hormone, steroid, edema, male, estradiol, vehicle, neuroprotection, sex, estrogens, studies, following, receptors, effect, trials, receptor, may, administration, role, animals, females, allopregnanolone, clinical, actions, groups, also, ovariectomized, compared, mgkg, potential, mechanisms, nervous, group, p4, 17estradiol, days, increased	301

**FIG. 2.**
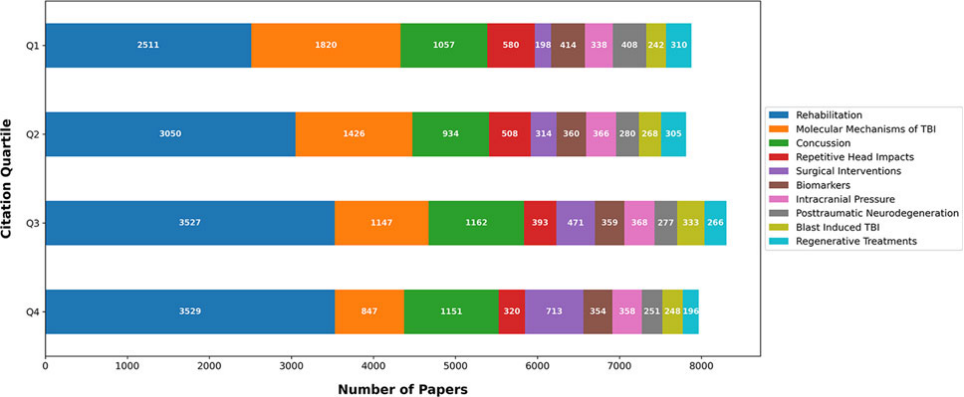
Citation quartiles for the top 10 topics. TBI, traumatic brain injury.

**FIG. 3.**
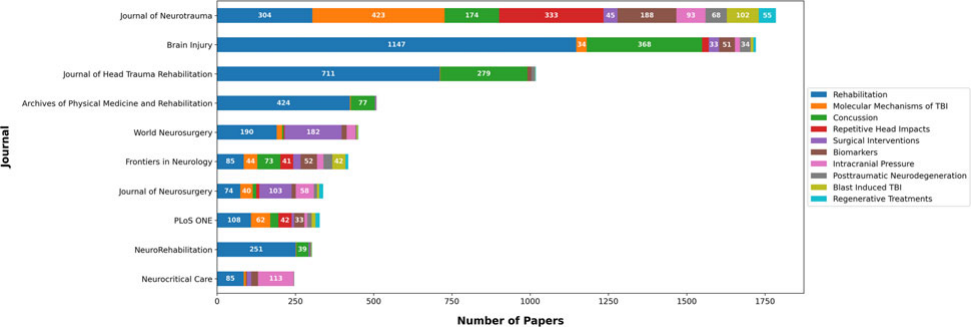
Topic distribution of articles by the top 10 journals. TBI, traumatic brain injury.

**FIG. 4.**
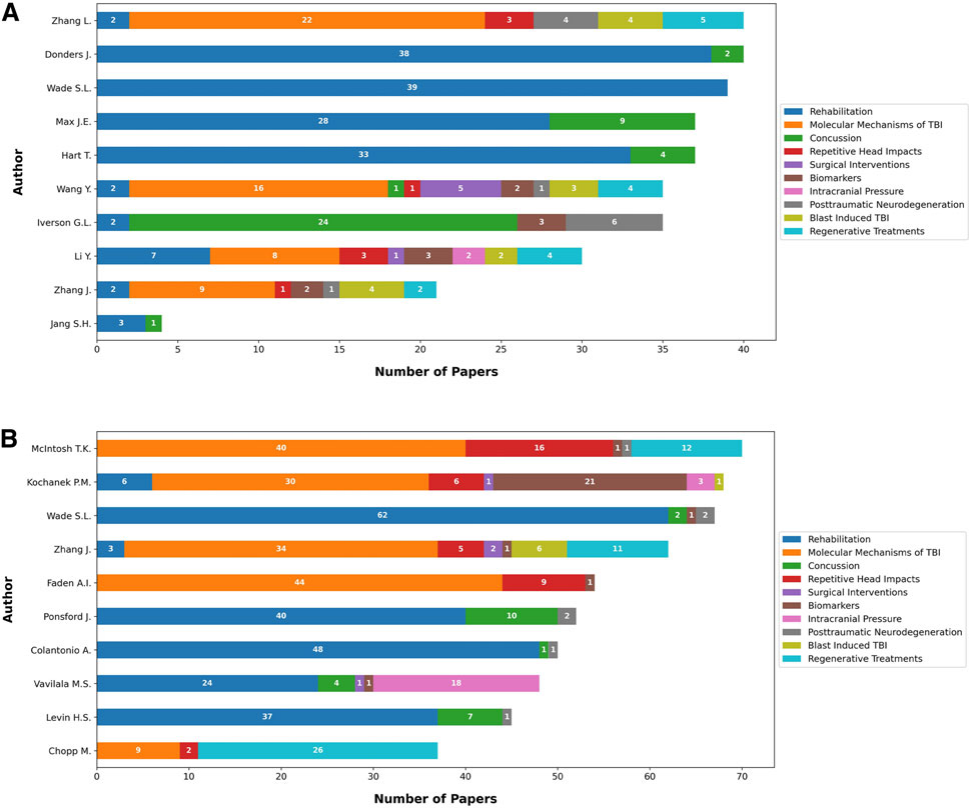
(**A**) Topic distribution of articles by the top 10 first authors. (**B**) Topic distribution of articles by the top 10 senior authors. TBI, traumatic brain injury.

Figure 2 illustrates an analysis of citation quartiles for the top 10 topics, offering insights into the impact and recognition of these topics within the research community. Figure 3 showcases the distribution of the number of articles related to each of the top 10 topics across different journals. Notably, the *Journal of Neurotrauma* published the highest number of articles related to the topic “Molecular Mechanisms of TBI,” whereas the journal *Brain Injury* predominantly published articles related to “Rehabilitation.” Figure 4A depicts the distribution of topics among the articles authored by the top 10 first authors. For example, L. Zhang authored 40 articles across the top 10 topics, 22 of which were associated with the topic “Molecular Mechanisms of TBI.” Figure 4B presents a similar analysis for the top 10 senior authors, defined by the last author. For instance, T.K. Mcintosh served as the senior author for 70 articles among the top 10 topics, 40 of which were related to the topic “Molecular Mechanisms of TBI.”

### Overall trends

By using linear regression models for the analysis of topic probabilities, we uncovered significant trends within our data set. Our analysis revealed that “Posttraumatic Headache,” “Brain Connectivity,” and “Mental Status” emerged as topics of steadily growing interest and emphasis, as evidenced by their consistently increasing slopes. In contrast, “Posttraumatic Sleep,” “Biopsychosocial Factors,” and “Sensory Functions” exhibited negative slopes, indicating a gradual decline in their representation within the literature and identifying them as cold topics.

**FIG. 5.**
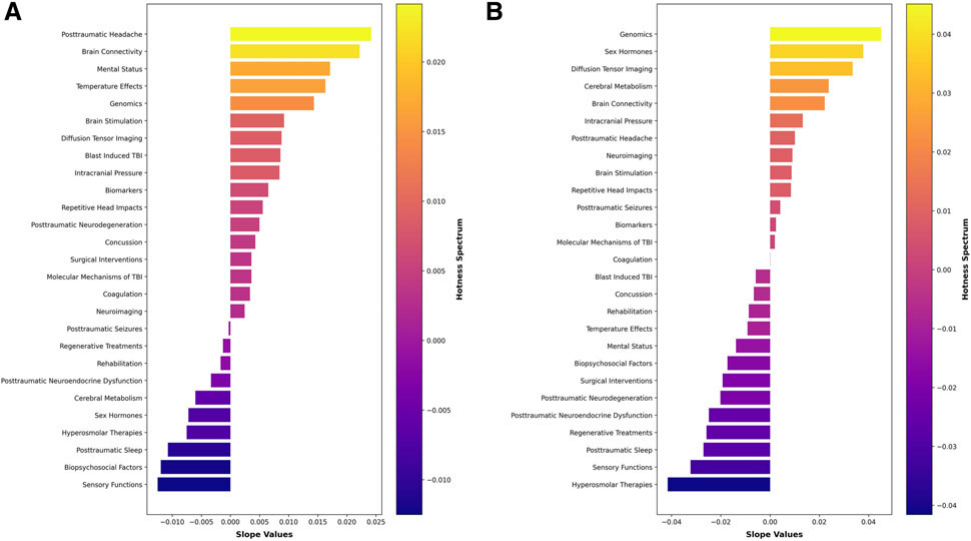
(**A**) Color-coded bar chart of hot and cold topics spanning from the inception of the first publication to the most recent publication date. (**B**) Color-coded bar chart of hot and cold topics, specifically in the current decade. TBI, traumatic brain injury.

Figure 5A provides a visual representation of these trends with a bar chart of color-coded bars. The length of each bar corresponds to the magnitude of the slope values, with the color spectrum ranging from darker shades of purple-blue (colder topics) to brighter shades of yellow-orange (hotter topics). This color gradient effectively communicates the trends, offering a straightforward and easily comprehensible visualization of the topic trends.

**FIG. 6.**
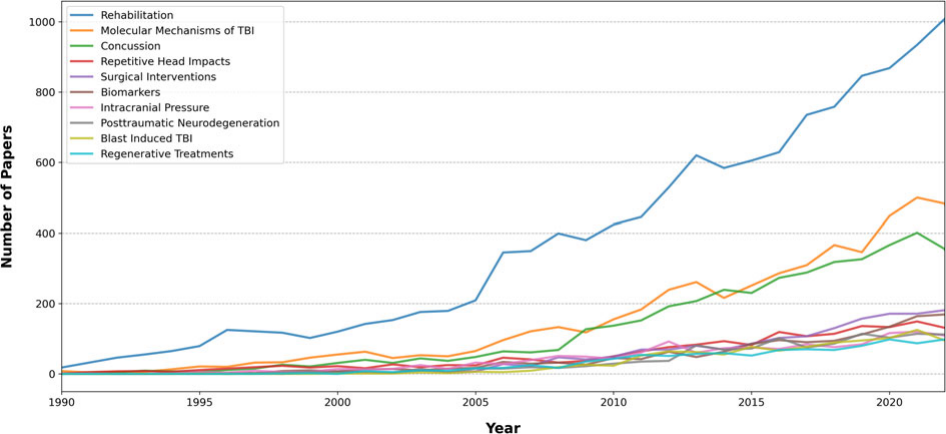
Number of articles for the top 10 topics plotted against publication year. TBI, traumatic brain injury.

For a clearer understanding of the temporal changes in the top 10 topics, Figure 6 presents a line plot that showcases the number of articles associated with each topic plotted against the publication year. This visualization offers valuable insights into the evolving significance and relevance of each topic throughout the years.

### Trends in the current decade

A focused analysis of trends within the current decade revealed that “Genomics,” “Sex Hormones,” and “Diffusion Tensor Imaging” emerged as the hottest topics. These areas have seen a substantial increase in representation, indicating a shift in research focus toward these subjects. In contrast, the topics of “Posttraumatic Sleep,” “Sensory Functions,” and “Hyperosmolar Therapies” were identified as the coldest topics of the decade, suggesting a decreased emphasis on these areas despite their earlier significance (Fig. 5B).

## Discussion

Our study used the advanced NLP-based topic modeling method, BERTopic, to characterize the publication landscape of TBI research, with a focus on identifying prominent research topics and tracking topical trends over time. The study highlighted numerous insights into the dynamic landscape of TBI research, demonstrating the potential of NLP as a tool to streamline research synthesis and provide an efficient means to navigate and understand the complex body of academic literature within the field.

The use of BERTopic in our study offers several notable advantages over traditional topic modeling methodologies, such as Latent Dirichlet Allocation (LDA).^14–16^ Unlike LDA, which relies primarily on word frequency to discern topics,^17^ BERTopic utilizes the advanced language comprehension capabilities of transformer-based models like BERT. This approach allows it to effectively capture context and semantics within text,^9^ leading to more accurate and coherent topic classifications. Additionally, BERTopic's unique combination of UMAP for dimensionality reduction and HDBSCAN for clustering enables it to detect topics of varying densities, a task that often poses a challenge for LDA. This ability allows for a more nuanced understanding of the structure of our text data. Further, BERTopic's capability to identify and handle outliers, which could otherwise skew topic modeling outcomes in methodologies like LDA, enhances the robustness of our results. Thus, the superior accuracy, flexibility, and robustness of BERTopic underscore its selection for our study and position it as a significant advancement over traditional topic modeling methodologies.

Our analysis yielded 27 distinct topics within TBI literature, spanning a broad range of themes and thus highlighting the diverse nature of research within the field over the past 30 years. We noticed that some topics were represented more frequently than others, signifying the varying areas of interest and focus within the TBI research community. The 10 most frequently occurring topics covered a wide array of TBI research, ranging from the molecular mechanisms of TBI to surgical interventions. The distribution of these topics across citation quartiles, first authors, and senior authors provided valuable insights into the popularity and impact of various research themes. These insights could guide researchers in pinpointing the areas of research that attract the most attention and have the greatest impact within the field.

Our trend analysis for the current decade highlights a significant shift in TBI research priorities, with increased emphasis on “Genomics,” “Sex Hormones,” and “Diffusion Tensor Imaging.” The growing focus on “Genomics” can be attributed to an evolving understanding of its role in the clinical outcomes of TBI.^18^ The rising interest in “Sex Hormones” possibly stems from research findings that indicate distinct effects of TBI on the concentration of sex-steroid hormones in male and female patients, with plasma levels of testosterone being predictive of recovery from unconsciousness after TBI in male patients.^19^ Last, the heightened emphasis on diffusion tensor imaging (DTI), an advanced magnetic resonance imaging technique providing insights into the brain's neuroanatomical connectome, likely reflects the sustained advancements in DTI over the past three decades.^20^ The artificial intelligence-based topic modeling approach utilized in our study presents a valuable tool for assessing the specialization of TBI journals and tracking the evolution of topics over time. This perspective is highly significant for academics, research funding bodies, and journal publishers. Understanding the patterns and frequency of topics within a specific journal aids researchers in determining the most suitable platform for their work, ensuring that it aligns with the journal's thematic focus.

Trends in research are inextricably linked to trends in funding and federal priorities given that funding drives thematic focus. By depicting the emergence, evolution, and decline of topics historically, our approach provides a contextual understanding of current research trends and might offer predictive insights into future research directions. It is important to note, however, that hot topics are not necessarily the topics to garner the most attention or have the greatest impact. They may also signal an area to avoid, given that it may be over-represented and/or saturated already. Researchers desiring to stand out may consider examining colder topics. Consequently, our methodology holds the potential for adoption for data-driven insights into the academic publishing landscape, ultimately enhancing the strategic positioning and effectiveness of scholarly research.

Like any research, our study has key strengths and limitations. To our knowledge, our study is the first to utilize NLP-driven topic modeling for analyzing research trends in the field of TBI. By adopting the BERTopic methodology, we were able to conduct a comprehensive and efficient review of a vast body of TBI literature, uncovering valuable insights that would be challenging to obtain using conventional review methods. Further, our approach enabled the identification of both historical and more recent patterns in TBI research, providing valuable insights into the constantly evolving research landscape. As for limitations, the effectiveness of our topic modeling technique relies on the reliability and completeness of the available metadata, which can vary across different articles. Also, our trend analysis was limited to linear trends, potentially falling short of capturing the intricate nature of TBI research's progression in its entirety.

## Conclusion

Our study, utilizing BERTopic, provided valuable insights into the field of TBI research, uncovering key trends and shifting areas of focus. We explored a diverse range of topics within the field, from “Rehabilitation” to “Blast Induced TBI.” The trend analysis conducted as part of our study revealed emerging hot topics such as “Posttraumatic Headache,” “Brain Connectivity,” and “Mental Status,” highlighting a growing interest in these areas. On the other hand, we noticed a relative decrease in research focus on topics like “Posttraumatic Sleep,” “Biopsychosocial Factors,” and “Sensory Functions.” Our study highlights the dynamic nature of TBI research and provides a crucial perspective on its current landscape and potential future directions.

## Supplementary Material

Supplemental data

Supplemental data

## Transparency, Rigor, and Reproducibility Summary

The study and analysis plan were not formally pre-registered, but the team member with primary responsibility for the analysis certifies that the analysis plan was pre-specified. The methodology that was followed in this study precludes a straightforward *a priori* estimate of power. A total of 62,181 articles were obtained from Scopus. Refining the search with criteria, 12,690 articles were excluded. An additional 1670 articles were excluded because of missing abstracts; 47,821 articles remained, of which 42,422 were successfully categorized into 27 distinct topics. The remaining 5399 documents, representing 11.3% of the total, were considered outliers that did not distinctly align with any specific topic. The pre-processing and topic modeling steps were described in detail in the main text. No replication or external validation studies have been performed or are planned/ongoing at this time to our knowledge. The source code for pre-processing and analyzing the data is available upon a reasonable request to the corresponding author of this study. Mert Karabacak had full access to all the data in the study and takes responsibility for the integrity of the data and the accuracy of the data analysis. The authors agree to provide the full content of the manuscript on request by contacting the authors of this study.

## Abbreviations Used

DTI: diffusion tensor imaging
LDA: Latent Dirichlet Allocation
NLP: natural language processing
TBI: traumatic brain injury
