# Supplementary material for: Exploiting Natural Language Processing to Unveil Topics and Trends of Traumatic Brain Injury Research
Source: Neurotrauma Rep. 2024 Mar 6;5(1):203–14. doi: 10.1089/neur.2023.0102 (PMC10924051; doi:10.1089/neur.2023.0102)
Supplement: Supplemental data [file Suppl_TableS1.docx]

**Supplementary Table 1.** Three representative documents for each topic.

| **Topic Label** | **Representative Document 1** | **Representative Document 2** | **Representative Document 3** |
| --- | --- | --- | --- |
| **Rehabilitation** | US population estimates of health and social outcomes 5 years after rehabilitation for traumatic brain injury | Clinical Deterioration and Neurocritical Care Utilization in Pediatric Patients With Glasgow Coma Scale Score of 9-13 After Traumatic Brain Injury: Associations With Patient and Injury Characteristics | The severe traumatic brain injury in Austria: Early rehabilitative treatment and outcome |
| **Molecular Mechanisms of TBI** | Apoptosis after traumatic brain injury | Activation of NLRP3 Is Required for a Functional and Beneficial Microglia Response after Brain Trauma | Activation of microglial N-methyl-D-aspartate receptors triggers inflammation and neuronal cell death in the developing and mature brain |
| **Concussion** | Time interval between concussions and symptom duration | Associations among PTSD and Postconcussive Symptoms in the Long-Term Impact of Military-Relevant Brain Injury Consortium-Chronic Effects of Neurotrauma Consortium Prospective, Longitudinal Study Cohort | The association of concussion history and symptom presentation in combat sport athletes |
| **Repetitive Head Impacts** | New method to induce mild traumatic brain injury in rodents produces differential outcomes in female and male Sprague Dawley rats | Tau reduction diminishes spatial learning and memory deficits after mild repetitive traumatic brain injury in mice | Cognitive Deficits Following Traumatic Brain Injury Produced by Controlled Cortical Impact |
| **Surgical Interventions** | Complications of decompressive craniectomy for traumatic brain injury | Outcome following decompressive craniectomy in severe head injury: Rashid Hospital experience | Functional Outcome After Primary Decompressive Craniectomy for Acute Subdural Hematoma in Severe Traumatic Brain Injury |
| **Biomarkers** | Utility of neuron-specific enolase in traumatic brain injury; relations to S100B levels, outcome, and extracranial injury severity | Early CSF and Serum S100B Concentrations for Outcome Prediction in Traumatic Brain Injury and Subarachnoid Hemorrhage | "S100b as a prognostic biomarker in outcome prediction for patients with severe traumatic brain injury |
| **Intracranial Pressure** | Invasive brain tissue oxygen and intracranial pressure (ICP) monitoring versus ICP-only monitoring in pediatric severe traumatic brain injury | Monitoring intracranial pressure in traumatic brain injury | Observations on the Cerebral Effects of Refractory Intracranial Hypertension After Severe Traumatic Brain Injury |
| **Posttraumatic Neurodegeneration** | Traumatic Brain Injury and Risk of Long-Term Brain Changes, Accumulation of Pathological Markers, and Developing Dementia: A Review | Clinical and neuropsychological profile of patients with dementia and chronic traumatic encephalopathy | Alzheimer's disease and chronic traumatic encephalopathy: Distinct but possibly overlapping disease entities |
| **Blast Induced TBI** | Significant head accelerations can influence immediate neurological impairments in a murine model of blast-induced traumatic brain injury | An assessment of blast modelling techniques for injury biomechanics research | Direct Observation of Low Strain, High Rate Deformation of Cultured Brain Tissue During Primary Blast |
| **Regenerative Treatments** | Therapeutic Application of Stem Cells in the Repair of Traumatic Brain Injury | Proliferation, migration, and differentiation of human neural stem/progenitor cells after transplantation into a rat model of traumatic brain injury | Stem cells: A promising candidate to treat neurological disorders |
| **Diffusion Tensor Imaging** | Diffusion tensor imaging in the corpus callosum in children after moderate to severe traumatic brain injury | Corpus callosum integrity and neuropsychological performance after traumatic brain injury: A diffusion tensor imaging study | White matter integrity and cognition in chronic traumatic brain injury: A diffusion tensor imaging study |
| **Coagulation** | Acute and delayed mild coagulopathy are related to outcome in patients with isolated traumatic brain injury | Early coagulopathy is an independent predictor of mortality in children after severe trauma | Factors associated with the development of coagulopathy after open traumatic brain injury |
| **Cerebral Metabolism** | Damage Control Resuscitation Supplemented with Vasopressin in a Severe Polytrauma Model with Traumatic Brain Injury and Uncontrolled Internal Hemorrhage | Arterial lactate above 2 mM is associated with increased brain lactate and decreased brain glucose in patients with severe traumatic brain injury | Hypertonic lactate to improve cerebral perfusion and glucose availability after acute brain injury |
| **Genomics** | Role of Long Noncoding RNAs in the Regulation of Cellular Immune Response and Inflammatory Diseases | The role of long noncoding RNA in traumatic brain injury | MicroRNA in central nervous system trauma and degenerative disorders |
| **Posttraumatic Seizures** | A review of seizures and epilepsy following traumatic brain injury | Seizures and the Role of Anticonvulsants After Traumatic Brain Injury | Epilepsy in head injury |
| **Neuroimaging** | Neuroimaging of traumatic brain injury | Based on a lecture at the annual meeting 2014 of the Swiss neurology society: Head and brain trauma: General aspects and neuroimaging | Early magnetic resonance imaging is unnecessary in patients with traumatic brain injury |
| **Biopsychosocial Factors** | The magnitude and correlates of alcohol and drug use before traumatic brain injury | Who responds better? Factors influencing a positive response to brief alcohol interventions for individuals with traumatic brain injury | Longitudinal Patterns of Alcohol Use Following Traumatic Brain Injury in an Active Duty and Young Veteran Military Sample: A VA TBI Model Systems Study |
| **Brain Connectivity** | Effects of mild traumatic brain injury and post-traumatic stress disorder on resting-state default mode network connectivity | Default mode network functional and structural connectivity after traumatic brain injury | Alterations of connectivity patterns in functional brain networks in patients with mild traumatic brain injury: A longitudinal resting-state functional magnetic resonance imaging study |
| **Sensory Functions** | Vestibular and balance function in veterans with chronic dizziness associated with mild traumatic brain injury and blast exposure | Visual problems associated with traumatic brain injury | Visual-vestibular processing deficits in mild traumatic brain injury |
| **Posttraumatic Neuroendocrine Dysfunction** | Pituitary pathology in traumatic brain injury: a review | Traumatic brain injury induced hypothalamic-pituitary dysfunction: A paediatric perspective | Occurrence of pituitary dysfunction following traumatic brain injury |
| **Mental Status** | From unresponsive wakefulness to minimally conscious PLUS and functional locked-in syndromes: Recent advances in our understanding of disorders of consciousness | Which behaviours are first to emerge during recovery of consciousness after severe brain injury? | Disorders of Consciousness |
| **Temperature Effects** | Therapeutic hypothermia in brain injuries and related diseases | Is there still a role for hypothermia in neurocritical care? | Effect of 35C hypothermia on intracranial pressure and clinical outcome in patients with severe traumatic brain injury |
| **Posttraumatic Sleep** | Characterizing self-reported sleep disturbance after mild traumatic brain injury | Sleep problems in children | Changes in sleep patterns following traumatic brain injury: A controlled study |
| **Brain Stimulation** | Noninvasive brain stimulation for cognitive rehabilitation following traumatic brain injury: a systematic review | Preliminary guidelines for safe and effective use of repetitive transcranial magnetic stimulation in moderate to severe traumatic brain injury | Electrical stimulation methods and protocols for the treatment of traumatic brain injury: a critical review of preclinical research |
| **Hyperosmolar Therapies** | Hypertonic saline versus mannitol for the treatment of increased intracranial pressure in traumatic brain injury | Comparison of Equimolar Doses of Mannitol and Hypertonic Saline for the Treatment of Elevated Intracranial Pressure after Traumatic Brain Injury A Systematic Review and Meta-Analysis | Mannitol or hypertonic saline in the setting of traumatic brain injury: What have we learned? |
| **Posttraumatic Headache** | Characterization of headache after traumatic brain injury | Natural history of headache after traumatic brain injury | Clinical Perspectives on Headache After Traumatic Brain Injury |
| **Sex Hormones** | Progesterone and neuroprotection | Progesterone in the brain: Hormone, neurosteroid and neuroprotectant | Progesterone protects against lipid peroxidation following traumatic brain injury in rats |
