# Supplementary figures and images for: Exploiting Natural Language Processing to Unveil Topics and Trends of Traumatic Brain Injury Research
Source: Neurotrauma Rep. 2024 Mar 6;5(1):203–14. doi: 10.1089/neur.2023.0102 (PMC10924051; doi:10.1089/neur.2023.0102)

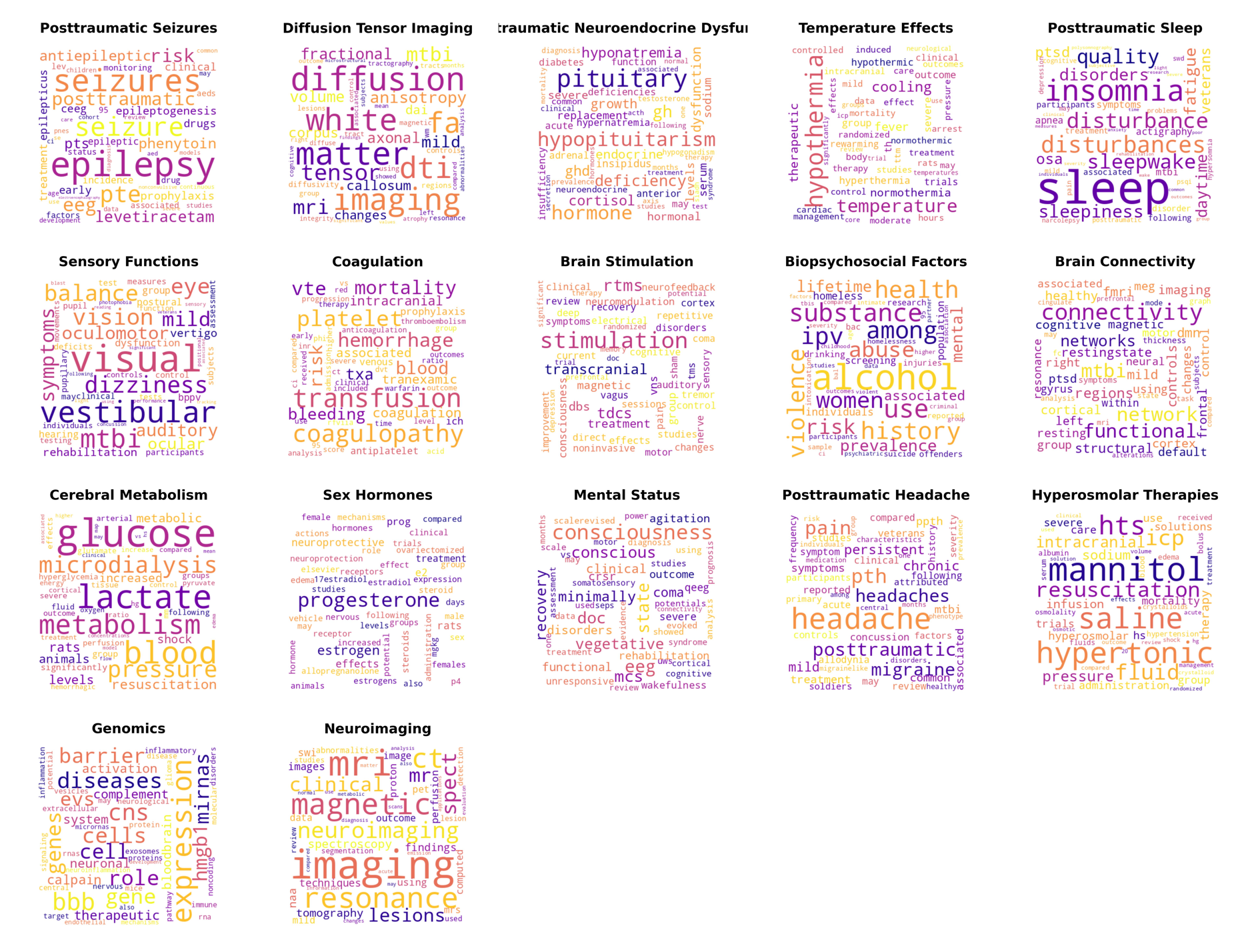
**Supplementary Figure 1.** Word clouds of remaining topics.

Supplement: Supplemental data [file Suppl_FigureS1.docx]
